# Atypical Presentations of Collagenous Gastritis Mimicking Celiac Sprue

**DOI:** 10.1155/2023/4073588

**Published:** 2023-02-13

**Authors:** Mark G. Evans, Jack P. Guccione, Anthony Crymes, Xiaodong Li, Cary A. Johnson, Vishal S. Chandan, Yuxin Lu

**Affiliations:** ^1^Caris Life Sciences, Phoenix, AZ, USA; ^2^Los Angeles County Medical Examiner-Coroner, Los Angeles, CA, USA; ^3^Department of Medicine, Keck School of Medicine of the University of Southern California, Los Angeles, CA, USA; ^4^Kaiser Permanente Redwood City Medical Center, Redwood, CA, USA; ^5^Department of Pathology and Laboratory Medicine, University of California Irvine (UCI) Medical Center, Orange, CA, USA; ^6^CellNetix Pathology and Laboratories, Tacoma, WA, USA

## Abstract

Collagenous gastritis has been reported as a rare cause of nausea, diarrhea, weight changes, and early satiety in female patients. Here, we describe two women aged 43 and 71 years who presented with similar symptoms. Gastric biopsies from both individuals showed thickened, irregular subepithelial collagen bands (>10 *μ*m). The pathogenesis of collagenous gastritis is poorly understood, but it may be the presenting symptom for many underlying autoimmune conditions. In particular, there is a well-established connection between collagenous disorders of the gastrointestinal tract and celiac sprue, Sjögren syndrome, and lymphocytic colitis; however, none of these conditions had been diagnosed in our patients. The older woman had incidentally discovered hypogammaglobinemia and IgA deficiency, whereas the younger woman suffered from fibromyalgia. Although a gluten-free diet and budesonide have been effective in some cases, there is no standardized therapy for collagenous gastritis. Our patients trialed diet modification and have required no additional medical interventions.

## 1. Introduction

Collagenous gastritis (CG) was first described by Colletti and Trainer in 1989 as a focal thickening of subepithelial collagen in the stomach biopsy of a 15-year-old girl with gastrointestinal (GI) bleeding and abdominal discomfort [1]. Subsequently, less than 100 cases have been reported in the medical literature. This condition generally features irregular collagen deposition greater than 10 *μ*m in thickness within the gastric lamina propria. It is most prevalent in young adult women. Two clinical subtypes have been reported based on age [2]. The pediatric patients typically present with idiopathic abdominal pain and GI bleeding, whereas diarrhea, nausea, weight changes, and associated autoimmune conditions are more common in adult populations. In particular, CG has been associated with Sjögren syndrome, celiac sprue, and lymphocytic colitis [3]. Here we present atypical presentations of CG occurring concurrently with fibromyalgia and hypogammaglobinemia in two adult women, who were successfully managed with low-gluten diets.

## 2. Case 1

A 71-year-old woman had experienced nausea, vomiting, progressive weight loss, and early satiety for approximately 5 years. Her past medical history was notable for hyperlipidemia, osteoarthritis, and longstanding thrombocytosis treated with hydroxyurea. The patient first noticed her symptoms after being hospitalized for injuries sustained during a motor vehicle accident. She experienced several weeks of nausea and vomiting, resulting in a 20-pound weight loss. Her GI upset subsided, but over the following several years, she experienced chronic early satiety and progressive weight loss of an additional 80 pounds. The patient also endorsed alternating episodes of diarrhea and constipation but denied abdominal pain, hematemesis, melena, or rectal bleeding. However, she presented with anemia and blood hemoglobin of 9.5 g/dL.

**Figure 1:**
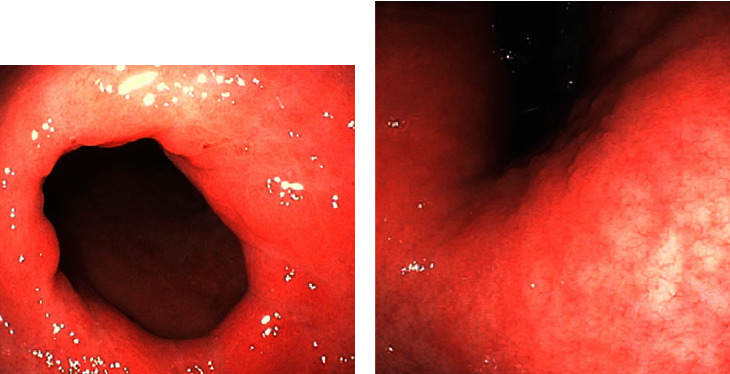
EGD/colonoscopies performed on the patients revealed hypertrophic gastric mucosa ((a) case 1, pylorus), as well as erythema, vascular congestion, and surface nodularity ((b) case 2, antrum).

**Figure 2:**
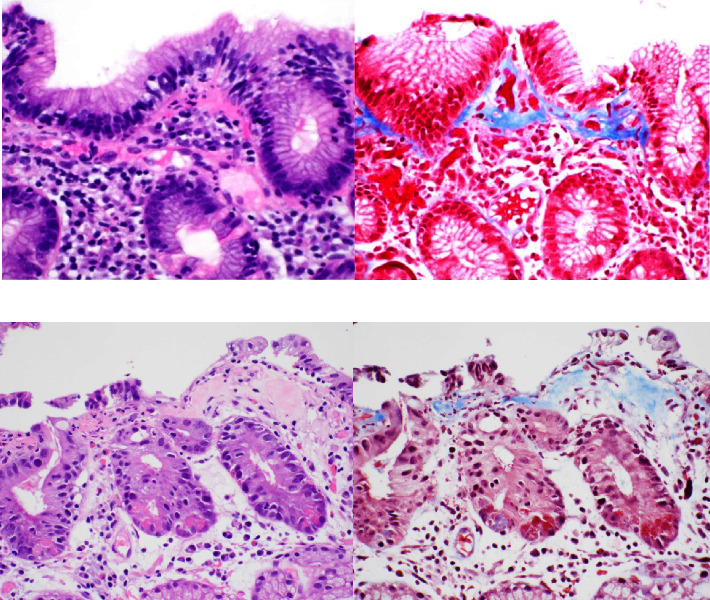
Hematoxylin and eosin staining of the patients' gastric biopsies demonstrates reactive lymphoid infiltrates and prominent bands of collagen within the lamina propria ((a) case 1; (b) case 2), 400x magnification. Masson's trichrome staining highlights the collagenous thickening >10 *μ*m, 400x magnification.

A gastric emptying study was unremarkable. The patient then underwent esophagogastroduodenoscopy (EGD) and endoscopic ultrasound (EUS) with gastric biopsies. The duodenum was not sampled, as it featured no endosonographic abnormalities. Her stomach antrum and body demonstrated hypertrophic, nodular-appearing mucosa (Figure 1(a)), diagnosed microscopically as atrophic gastritis with moderate activity and diffuse intestinal metaplasia. Moreover, the body biopsies showed patchy thickening of collagen bands up to 12 *μ*m within the lamina propria, as well as entrapped capillaries and surface epithelial disruption (Figure 2(a)). The overall findings were with consistent CG, but clinical and serologic correlation was required in order to exclude celiac sprue and autoimmune gastritis.

Incidentally, the patient was found to have hypogammaglobulinemia and specifically low levels of serum IgG and IgA (616 and 27 mg/dL, respectively). Her workup for celiac sprue and autoimmune gastritis was unrevealing, with normal testing for iron, anti-TTG/gliadin antibodies, and gastrin. However, in the setting of hypogammaglobulinemia, these results could not definitively rule out celiac sprue. Duodenal biopsy was considered but was not performed, as HLA-D2 and HLA-DQ8 testing were negative. Therefore, the patient was advised to try a gluten-free diet. Her early satiety resolved, she gained weight, and her hemoglobin improved up to 13.2 g/dL. And although the patient was recommended to repeat EGD/colonoscopy, she did not pursue additional care within the first year of follow-up.

## 3. Case 2

A 43-year-old woman with migraines, anxiety, and a vague history of irritable bowel syndrome complained of excessive postprandial pharyngeal secretions for six months, which were associated with cough, nausea, and vomiting. She also endorsed abdominal pain following ingestion of spicy foods but denied hematemesis, melena, weight changes, or rectal bleeding. A *Helicobacter pylori* breath test was negative, and she was empirically treated for gastroesophageal reflux disease with rabeprazole, resulting in the resolution of only her abdominal discomfort.

The patient then underwent EGD and colonoscopy, which demonstrated mild reflux esophagitis, a sessile serrated adenoma of the cecum, a tubular adenoma of the sigmoid colon, as well as erythematous, congested, and nodular gastric mucosa (Figure 1(b)). Moreover, random biopsies of the stomach antrum and body showed moderate chronic active gastritis in addition to increased intraepithelial lymphocytes and focal thickening of subepithelial collagen bands, up to 10 *μ*m (Figure 1(b)). These findings were most consistent with CG.

Allergy testing and a workup for celiac sprue and autoimmune gastritis, including antigliadin antibody, antitransglutaminase (TTG) antibody, antiparietal cell antibody, and intrinsic factor analysis, were unremarkable. Human leukocyte antigen (HLA) testing was not performed, but her duodenal biopsy had also shown no disruption of normal villous architecture. She was ultimately recommended to avoid nonsteroid anti-inflammatory drugs (NSAIDs) and to begin a low-gluten diet, resulting in complete symptom resolution. One and one-half years later, the patient developed diffuse myalgias. Extensive rheumatologic laboratory testing provided no evidence for a connective tissue disease or inflammatory myopathy. Instead, her symptoms were most consistent with myofascial pain syndrome/fibromyalgia, which has been successfully managed by acupuncture and physical therapy. At four years of follow-up, the patient continued to deny gastrointestinal complaints despite unchanged gastric mucosal findings observed by EGD/colonoscopy.

## 4. Discussion

We describe two atypical presentations of CG in female patients with symptoms of nausea, vomiting, weight changes, and early satiety. Gastric biopsies from both women showed histologic features of this condition. A microscopically thickened subepithelial collagen band with overlying gastric epithelial injury is a characteristic histologic feature of CG. Other findings include lymphoplasmacytosis in the lamina propria with eosinophils. Focal detachment or disruption of the surface epithelium and entrapped capillaries within the abnormally thickened collagen band are often appreciated. Studies have reported varying thickness of the collagen band, ranging from at least 10 *μ*m to greater than 100 *μ*m [4].

Previous reports have identified an association between GC and underlying autoimmune diseases such as celiac sprue, Sjögren syndrome, or lymphocytic colitis [5, 6]. One of the patients presented here also developed fibromyalgia. The connection between fibromyalgia and any collagenous disease of the GI tract has only been indirectly described in one case report [7]. These authors describe a woman of similar age and presenting symptoms as our patient diagnosed as collagenous colitis (CC). Further investigation and workup showed that she was erroneously diagnosed with fibromyalgia and had an underlying spondyloarthropathy with sacroiliitis on radiographic imaging. The authors suggest that fibromyalgia patients who develop diarrhea or (CC) should be carefully re-examined for any underlying inflammatory spinal or rheumatologic disease [7]. A small but growing number of collagenous colitis cases with underlying inflammatory spinal and rheumatologic diseases warrant collagenous colitis being included on the list of enteropathic peripheral and axial arthropathies [8–10]. In our other reported patient, low levels of serum IgA and IgG were identified, and celiac sprue could not be ruled out. The association between celiac and GC has been well-defined in the literature [11, 12]. One study reported that approximately 15% to 20% of patients carrying a diagnosis of collagenous colitis will also be diagnosed with celiac sprue [13].

In addition to its association with autoimmune conditions, CG in the adult population is often observed in the setting of concurrent CC [2]. In one review article of 60 CG cases, 9 had additional findings of extragastric collagen deposition in the colon, consistent with CC [14]. These two conditions are thought to share a similar, yet poorly understood pathogenesis. Some studies suggest the presence of chronic inflammation, fibroblast sheath abnormalities, and leakage of plasma proteins and fibrinogen, as in leaky gut syndrome [15]. Another common hypothesis describes an imbalance in extracellular matrix degradation and production of the different collagen types by a deficiency in metalloproteinases [16]. Food or drugs may also play a role in the etiology of collagenous GI diseases, as evidenced by their association with NSAIDS, histamine blockers, and proton pump inhibitors [13, 17, 18]. The underlying cause of diarrhea in adults with collagenous diseases is thought to be due to increased nitric oxide synthase activity that leads to diarrheal symptoms. While diarrhea is commonly observed in adults with CC and CG, neither of our patients reported this symptom. Moreover, endoscopy did not demonstrate visual colonic abnormalities in either woman, and extragastric collagen deposition was not observed within the duodenal biopsy of the younger individual. These findings reinforce the atypical presentations of our two patients, such that their chief complaints likely could be attributed to isolated CG and not to concurrent CC.

As is consistent with this case series, CG has a slight female predominance and presents with symptoms ranging from abdominal pain, diarrhea, weight loss, and GI bleeding [19]. Pediatric patients often demonstrate severe iron-deficiency anemia, likely due to the entrapped capillaries in the subepithelial collagen layer [19]. Whereas adults will typically develop watery chronic diarrhea, and their biopsies may also show extragastric collagen deposition, in the form of collagenous sprue of the small intestine [13, 20]. Endoscopically, the mucosa of adults and pediatric CG cases appears nodular and erythematous, with depressed areas surrounding the nodules corresponding to subepithelial collagen deposition seen histologically [14]. The natural history of the disease is variable; more than 80% of patients suffer from chronic, intermittent symptoms [19]. Less than 3% will have only one isolated episode.

Because the pathogenesis of CG is not well understood, there is no standardized treatment. The resolution of symptoms largely depends on the presence of any associated diseases [19]. Studies suggest that adults with CG and a concurrent celiac sprue may respond to a gluten-free or low diet; however, those with a collagenous sprue may respond poorly to dietary changes [6]. Some authors attribute GC to autoimmunity and the associated chronic inflammation that promotes subepithelial collagen deposition [21]. Specifically, patients develop hypersensitivity to ingested gluten, generating an inflammatory response. Consequently, eliminating dietary gluten removes antigenic stimuli. Of note, our patients did not require medications to manage their gastrointestinal complaints, as both completely responded to restricting their gluten intake—an outcome that has been reported in the medical literature [21]. However, when lifestyle modification is unsuccessful, budesonide, a glucocorticoid that exhibits good mucosal activity and limited systemic effects, has had utility in adults with collagenous gastritis and a concurrent collagenous colitis [22]. Bismuth, salicylates, prednisolone, mesalamine, cholestyramine, and antibiotics have been shown to provide varying degrees of symptom relief [19].

CG is part of a group of gastroenteritidies that share similar histologic findings characterized by thickened subepithelial collagen bands. Because it is not fully understood, it can be exceedingly difficult to treat if afflicted patients do not respond to gluten-free or low diets. Diagnosing an underlying celiac or autoimmune disease in patients with CG is paramount to hastening recovery from this unique and rare disease.

## Data Availability

No publicly archived datasets were utilized for this study, and all patient information is housed at the University of California, Irvine Medical Center electronic medical record.
